# Non-thermal plasma inhibits mast cell activation and ameliorates allergic skin inflammatory diseases in NC/Nga mice

**DOI:** 10.1038/s41598-019-49938-9

**Published:** 2019-09-18

**Authors:** Myung-Hoon Lee, Yun Sang Lee, Haeng Jun Kim, Chang Hak Han, Sung Un Kang, Chul-Ho Kim

**Affiliations:** 1 Department of Otolaryngology, School of Medicine, Ajou University, Suwon, Republic of Korea; 2 Department of Molecular Science and Technology, Ajou University, Suwon, Republic of Korea

**Keywords:** Electrophysiology, Inflammatory diseases

## Abstract

Non-thermal plasma (NTP) has many functional activities such as, sterilization, wound healing and anti-cancer activity. Despite of its wide spread biomedical application, the effect of NTP on immune cells and allergic response has not been well studied. In this study, we determined whether NTP suppresses mast cell activation, which is important for allergic response, and ameliorates an atopic dermatitis (AD)-like skin inflammatory disease in mice. Exposure to NTP-treated medium during mast cell activation inhibited the expression and production of IL-6, TNF-α and suppressed NF-κB activation. We also investigated whether NTP treatment ameliorates house dust mite (HDM)-induced AD-like skin inflammation in mice. NTP treatment inhibited increases in epidermal thickness and recruitment of mast cells and eosinophils, which are important cell types in AD pathogenesis. In addition, Th2 cell differentiation was induced by application of HDM and the differentiation was also inhibited in the draining lymph node of NTP-treated mice. Finally, the expression of AD-related cytokines and chemokines was also decreased in NTP-treated mice. Taken together, these results suggest that NTP might be useful in the treatment of allergic skin diseases, such as AD.

## Introduction

Atopic dermatitis (AD) is a common allergic skin disease, characterized by mast cell activation, eosinophilia, overexpression of cytokines and epithelial hyperplasia^1,2^. Although the etiology of AD is complex, many studies suggest that immune cells are involved in the pathogenesis of AD and uncontrolled immune response is one of the main causes of AD^3^. For example, in the acute phase of AD, Th2 cell percentage increases, and AD skin lesions express higher levels of Th2 cytokines and chemokines compared to normal skin^4,5^. In addition, the activation and infiltration of mast cells and eosinophils are also critical for AD^6^. By contrast, Th1 cells and their cytokines play a role in the chronic phase of AD^7^. Several treatments for AD, such as glucocorticoids, calcineurin inhibitors, phototherapy, and immunosuppressors (cyclosporine A), have been used^8^. However, these drugs and therapies cause many side effects including ulcers, thin skin, diabetes, depression, and slow-wound healing^9^. Therefore, the development of new treatments for AD without side effects imperative.

Plasma is referred to as the fourth state of matter and is composed of cations, anions, electrons and reactive species. During the last few decades, the field of plasma medicine has grown rapidly. Several studies show that plasma regulates various effects, such as anti-cancer^10–13^, anti-inflammation^14^, sterilization^15^, and tooth-bleaching effects^16^. Recently, we demonstrated that non-thermal plasma (NTP) treatment induces cancer cell death via AKT degradation^17^, promotes muscle regeneration in mice^18^, accelerates wound healing^19^, and inhibits psoriasis-like skin inflammation in mice^20^. Thus, plasma medicine is emerging in the biomedical field, and researchers are investigating whether plasma can be used for the treatment of various diseases. However, the anti-allergic effect of NTP is not well studied.

In this study, we investigated whether NTP inhibited mast cell activation and house dust mite (HDM)-induced AD-like skin inflammation in NC/Nga mice. Our results show that NTP treatment inhibited HDM-induced AD-like skin inflammation in NC/Nga mice. In addition, we demonstrated the inhibitory effect of liquid type plasma (LTP) on mast cell activation and the inflammation of keratinocytes, suggesting that the anti-allergic effect of NTP might at least partially result from the suppression of mast cell and keratinocyte activation.

## Results

### LTP treatment inhibits mast cell activation

**Figure 1:**
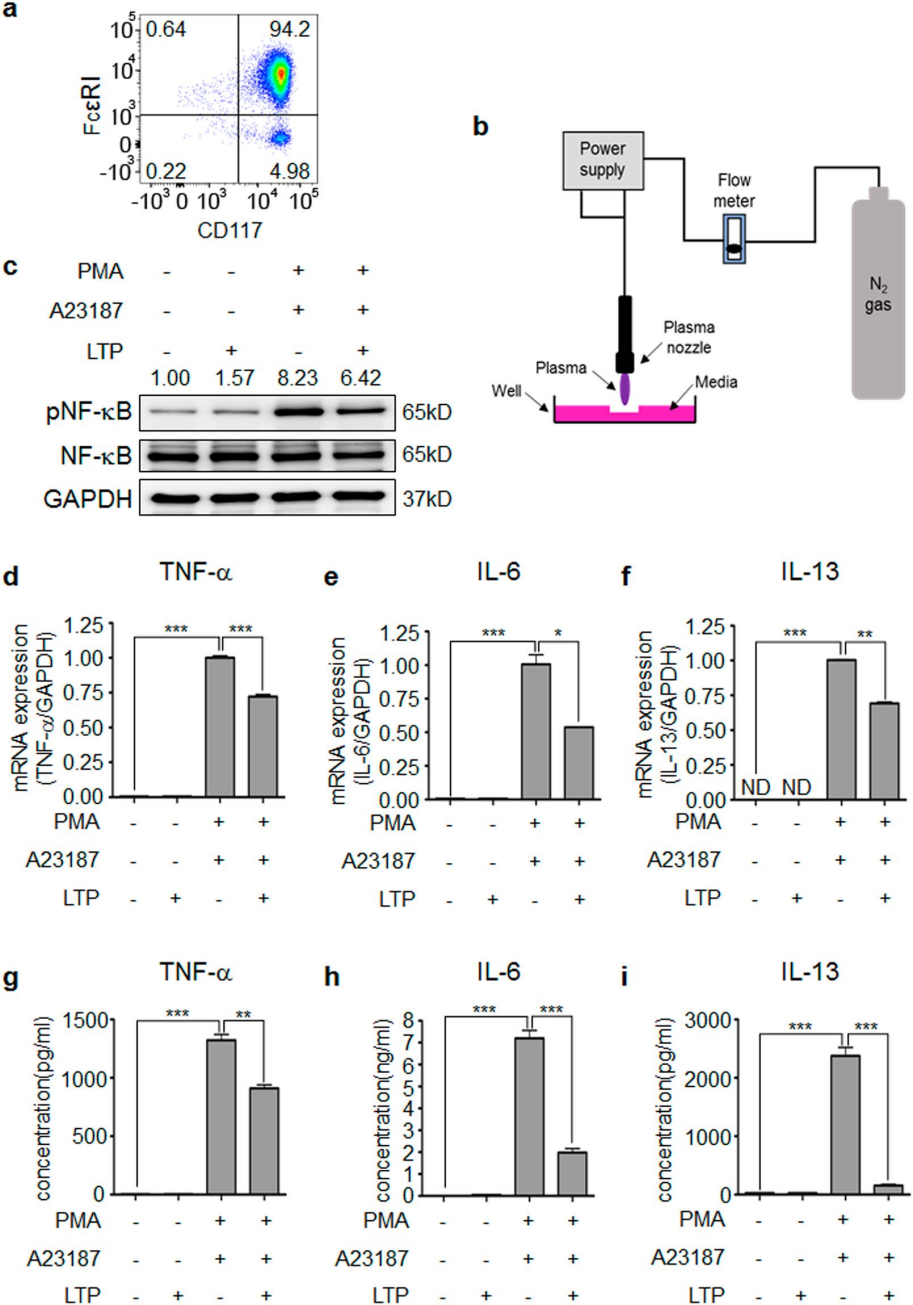
LTP inhibits mast cell activation. (**a**) Mast cells were generated from mouse bone-marrow derived lineage negative cells by culture with mIL-3 and mSCF for 8 weeks. The percentage of mast cells exceed 94%. (**b**) A schematic diagram showing the generation of LTP. Non-thermal plasma was treated for 60 sec per ml and used for *in vitro* experiments. (**c**) All Western blotting experiments were performed under the same condition. LTP treatment inhibits NF-κB activation in PMA/A23187-stimulated mast cells. LTP treatment inhibits (**d**) TNF-α, (**e**) IL-6, and (**f**) IL-13 expression in PMA/A23187-stimulated mast cells. LTP treatment reduced the secretion of (**g**) TNF-α, (**h**) IL-6, and (**i**) IL-13 by activated mast cells. *P < 0.05, **P < 0.01, ***P < 0.001.

We investigated whether plasma treatment inhibits mast cell activation, which is involved in an allergic response. To generate mouse primary mast cells, lineage-negative cells were isolated from bone marrow and cultured with mIL-3 and mSCF for 6 weeks (Fig. 1a) as described in Materials and Methods. LTP was made as shown in Fig. 1b and the mast cells were activated with PMA and A23187 with or without plasma-treated medium. As shown in Fig. 1c, NF-κB is activated in activated mast cells and LTP treatment inhibited the NF-κB activation. Activated mast cells also expressed increased proinflammatory cytokines such as TNF-α, IL-6 and IL-13, however, LTP treatment inhibited the increased levels of TNF-α, IL-6 and IL-13 expression (Fig. 1d–f). Consistently, the results of ELISA also showed that LTP treatment decreased the secretion of TNF-α, IL-6 and IL-13 (Fig. 1g–i). These results suggest that LTP treatment decreased the cytokine expression via inhibition of NF-κB activation and plasma treatment might inhibit the allergic response.

### NTP treatment ameliorate HDM-induced AD-like skin inflammation in mice

**Figure 2:**
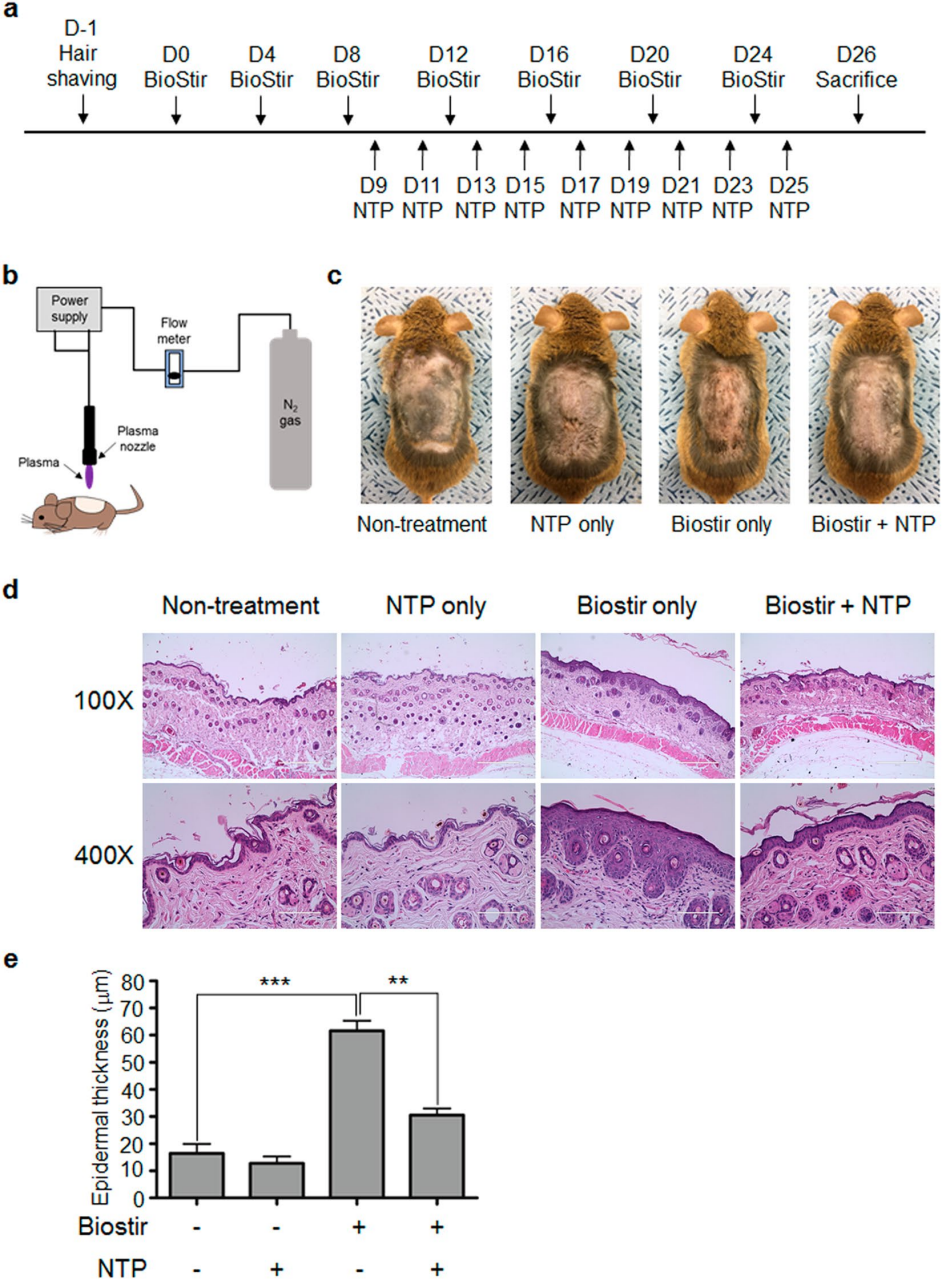
NTP treatment ameliorates HDM-induced AD-like skin inflammation in NC/Nga mice. (**a**) The experimental scheme for induction of HDM-induced AD-like skin inflammation and NTP treatment. (**b**) A schematic diagram describing treatment of mice with the NTP- producing machine. (**c**) NTP treatment inhibited AD-like skin inflammation in NC/Nga mice. (**d**) H&E staining of mouse back skin (**e**) NTP treatment reduced epidermal thickness. **P < 0.01, ***P < 0.001. Bar = 400 µm (100×), 100 µm (400×).

**Figure 3:**
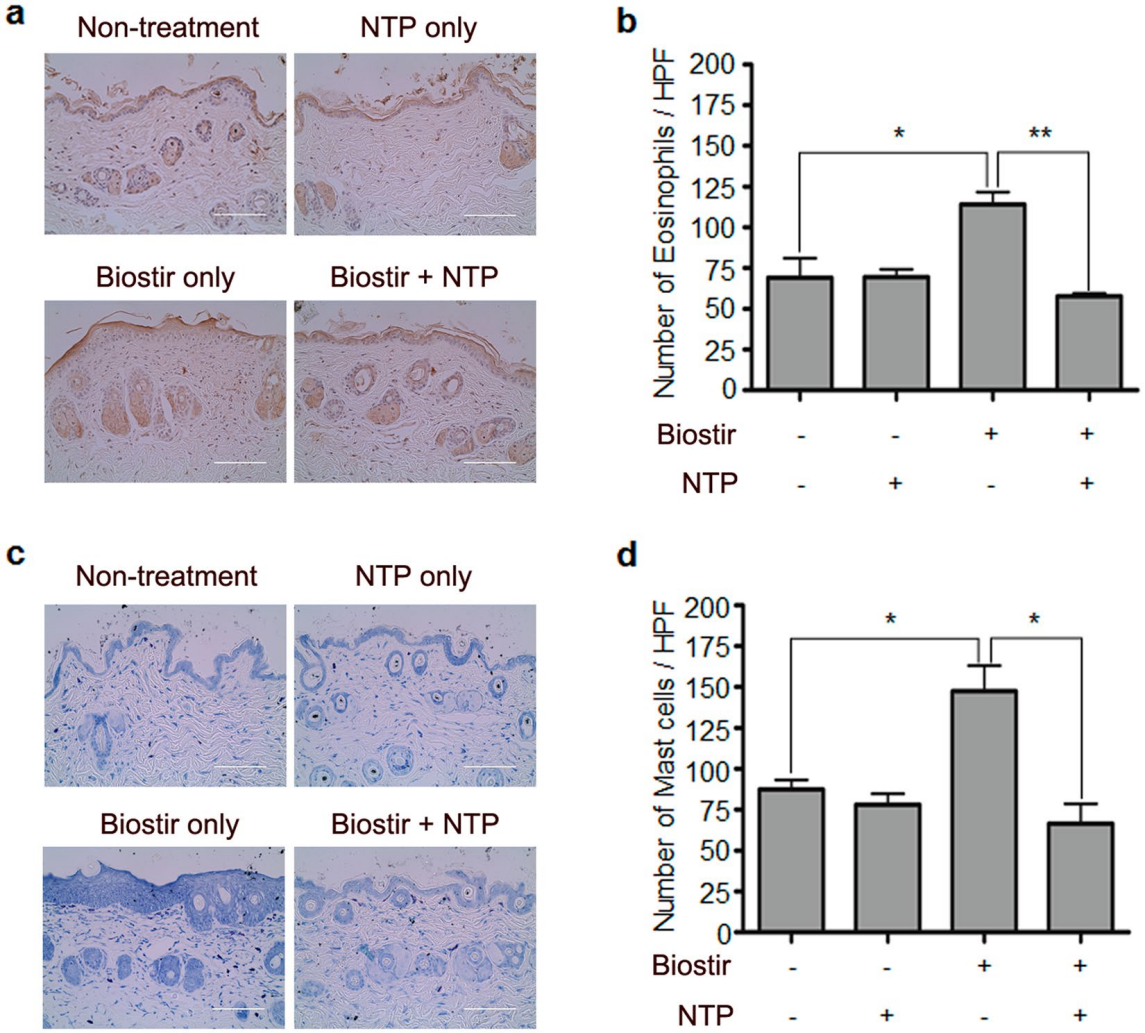
NTP treatment inhibits immune cell infiltration in the mouse skin. (**a**) NTP treatment inhibited eosinophil infiltration in HDM-induced AD-like mouse skin. (**b**) The number of infiltrated eosinophils is indicated as a bar graph. (**c**) NTP treatment inhibited mast cell infiltration in HDM-induced AD-like mouse skin. (**d**) The number of infiltrated mast cells is represented as a bar graph. *P < 0.05, **P < 0.01. Bar = 100 µm.

LTP treatment suppressed mast cell activation, suggesting that plasma could have anti-allergic effect. Thus, we investigated whether NTP could suppress AD-like skin inflammation in mice. After shaving back hair, a cream containing HDM (Biostir) was applied on NC/Nga mice every 4 days for 24 days, and NTP was treated every other day between day 9 and day 25. The mice were sacrificed on day 26 for analysis (Fig. 2a,b). Application of HDM induced AD-like skin inflammation in NC/Nga mouse skin and NTP treatment ameliorated the skin inflammation (Fig. 2c). Hematoxylin and Eosin (H&E) staining also revealed an increased epidermal thickness and immune cell infiltration in HDM-applied skin, while NTP treatment decreased the epidermal thickness and immune cell infiltration (Fig. 2d,e). We determined the number of eosinophils and mast cells among the infiltrated immune cells in the dermis because the cells are involved in the pathogenesis of AD. As shown in Fig. 3, exposure to HDM increased the infiltration of eosinophils (Fig. 3a,b) and mast cells (Fig. 3c,d). Meanwhile, NTP treatment suppressed the HDM-induced eosinophil and mast cell infiltration into the dermis. NTP treatment without HDM stimulation did not affect eosinophil and mast cell infiltration, suggesting that NTP treatment has no affect in normal skin.

### NTP treatment inhibits Th2 cell differentiation and AD-related gene expression in mice

**Figure 4:**
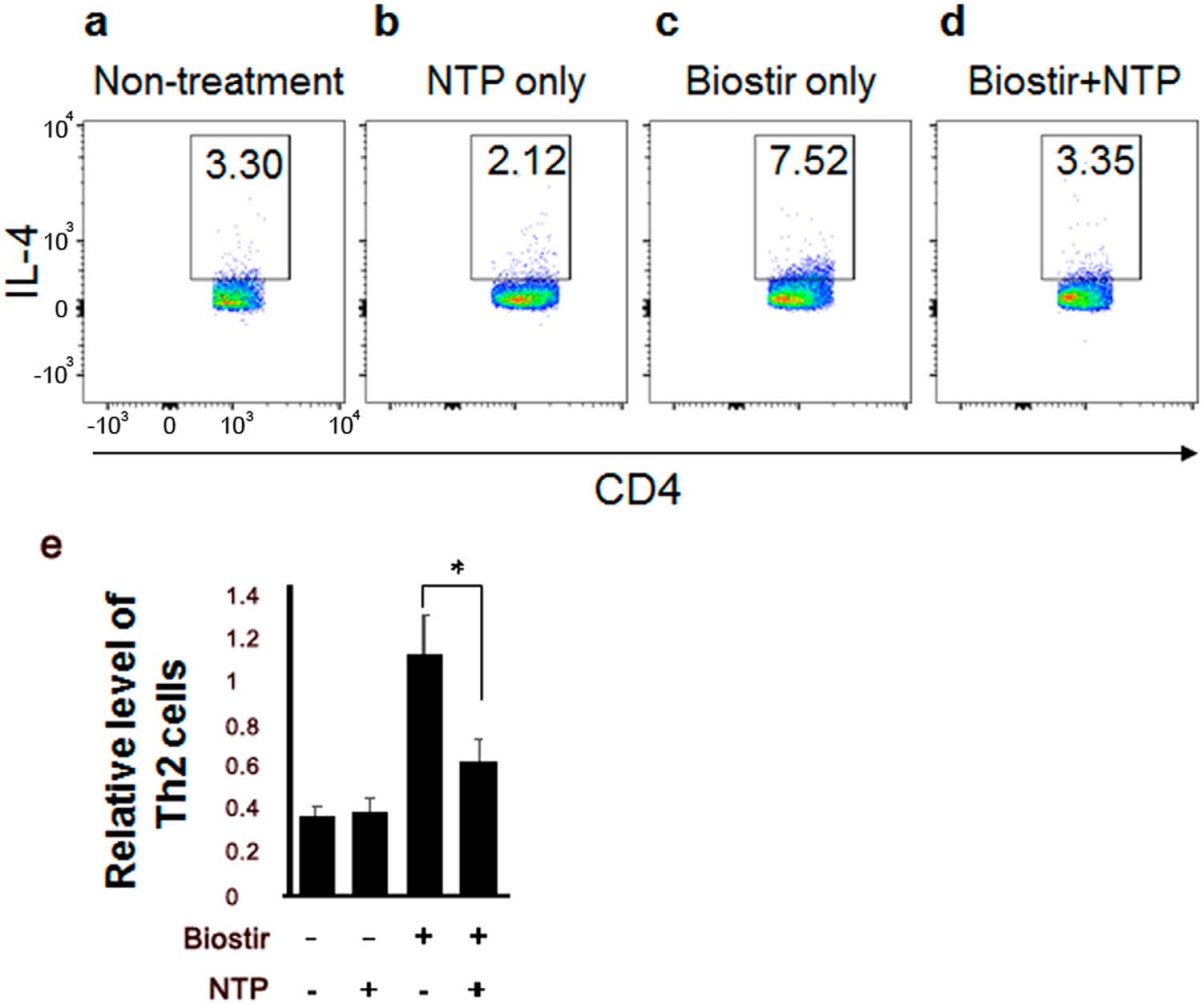
NTP treatment inhibited Th2 cell differentiation *in vivo*. Th2 cell differentiation in the draining lymph node in (**a**) non-treated and (**b**) NTP only-treated mice was similar. (**c**) HDM (Biostir)-induced Th2 cell differentiation in the mouse draining lymph node. (**d**) NTP treatment inhibited HDM-induced Th2 cell differentiation. (**e**) The Relative levels of Th2 cells are shown as a bar graph from four mice. *P < 0.05, **P < 0.01.

Th2 cells are one of the major cell types involved in AD pathogenesis. Thus, we determined whether NTP treatment affected Th2 cell differentiation in mice. As expected, the proportion of Th2 cells increased in the draining lymph node of HDM treated- mice (Fig. 4c,e). However, NTP treatment decreased the Th2 cell percentage (Fig. 4d,e), suggesting that NTP treatment inhibits Th2 cell differentiation. Th2 cell percentage in NTP-treated mice without HDM stimulation was not changed compared with that of NTP-untreated mice (Fig. 4a,b,e), implying that NTP treatment might not regulate unstimulated immune cells in normal mice. Consistently, treatment of LTP during Th2 cell differentiation attenuated the differentiation from naïve CD4 T cells to Th2 cells *in vitro* (Supplementary Fig. 1). These results imply that NTP treatment might inhibit Th2 cell differentiation

**Figure 5:**
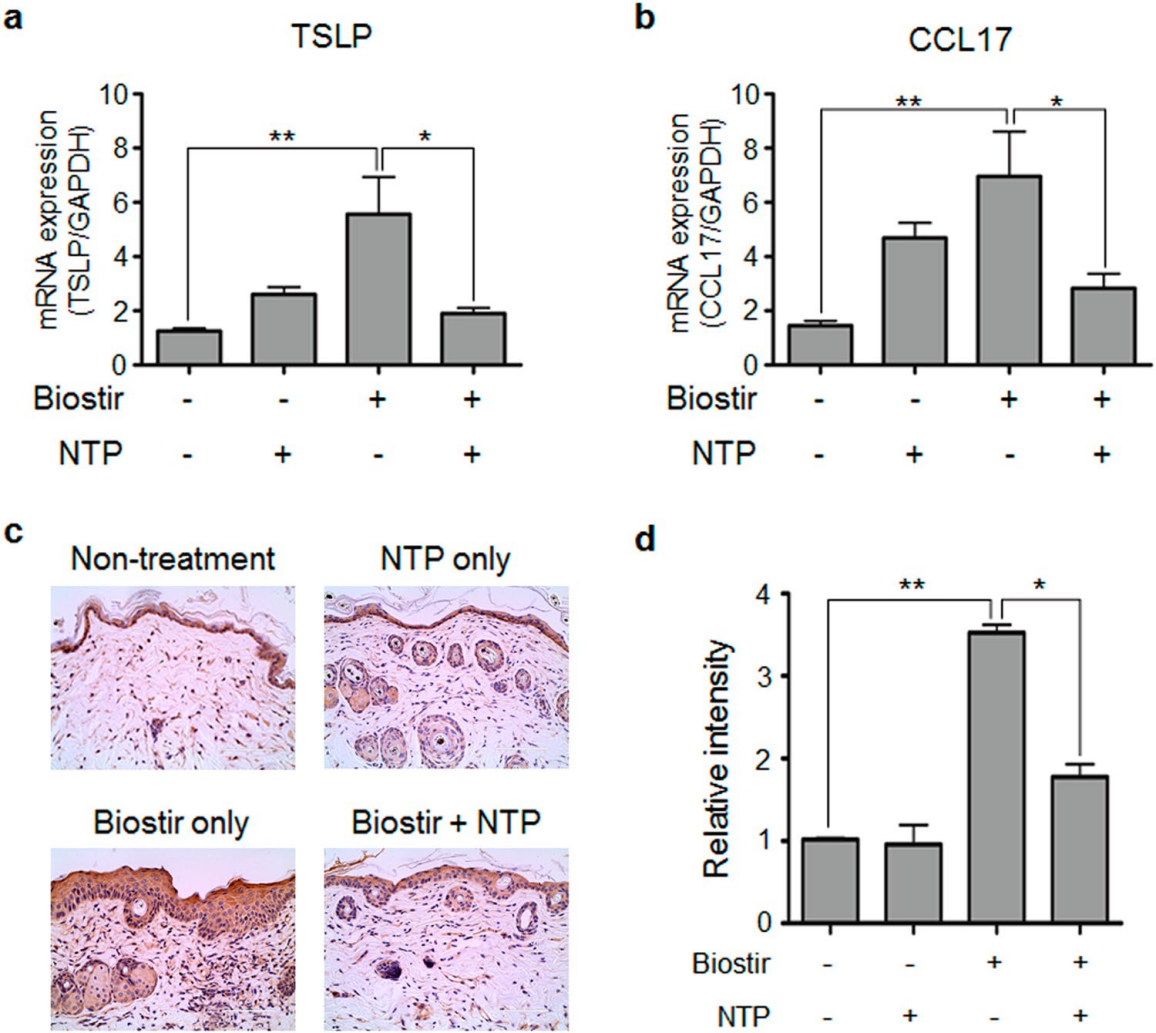
Cytokine and chemokine expression in the mouse skin. AD-related cytokine, (**a**) TSLP and (**b**) chemokine CCL17 expression increased in HDM-applied mouse skin and the expression was inhibited by NTP treatment. (**c**) NTP treatment reduced HDM-induced TSLP production in mouse skin. TSLP production was detected by IHC. (**d**) The level of TSLP is shown as a bar graph. *P < 0.05, **P < 0.01. Bar = 100 µm.

We also determined the AD-related cytokine and chemokine expression in mouse skin tissue. The levels of RNA expression of TSLP (Fig. 5a) and CCL17 (Fig. 5b) were increased in AD-induced mouse skin and were reduced by NTP treatment. In addition, using IHC analysis we confirmed that the production of TSLP, which is an important cytokine for AD pathogenesis, was also suppressed. As shown in Fig. 5c, the production of TSLP increased in HDM-applied mouse skin compared with that of HDM/NTP-treated mouse skin, which is consistent with real-time PCR results. The intensity of the TSLP signal was measured using ImageJ and shown as a graph (Fig. 5d).

### LTP treatment inhibits the inflammatory response in HaCaT

**Figure 6:**
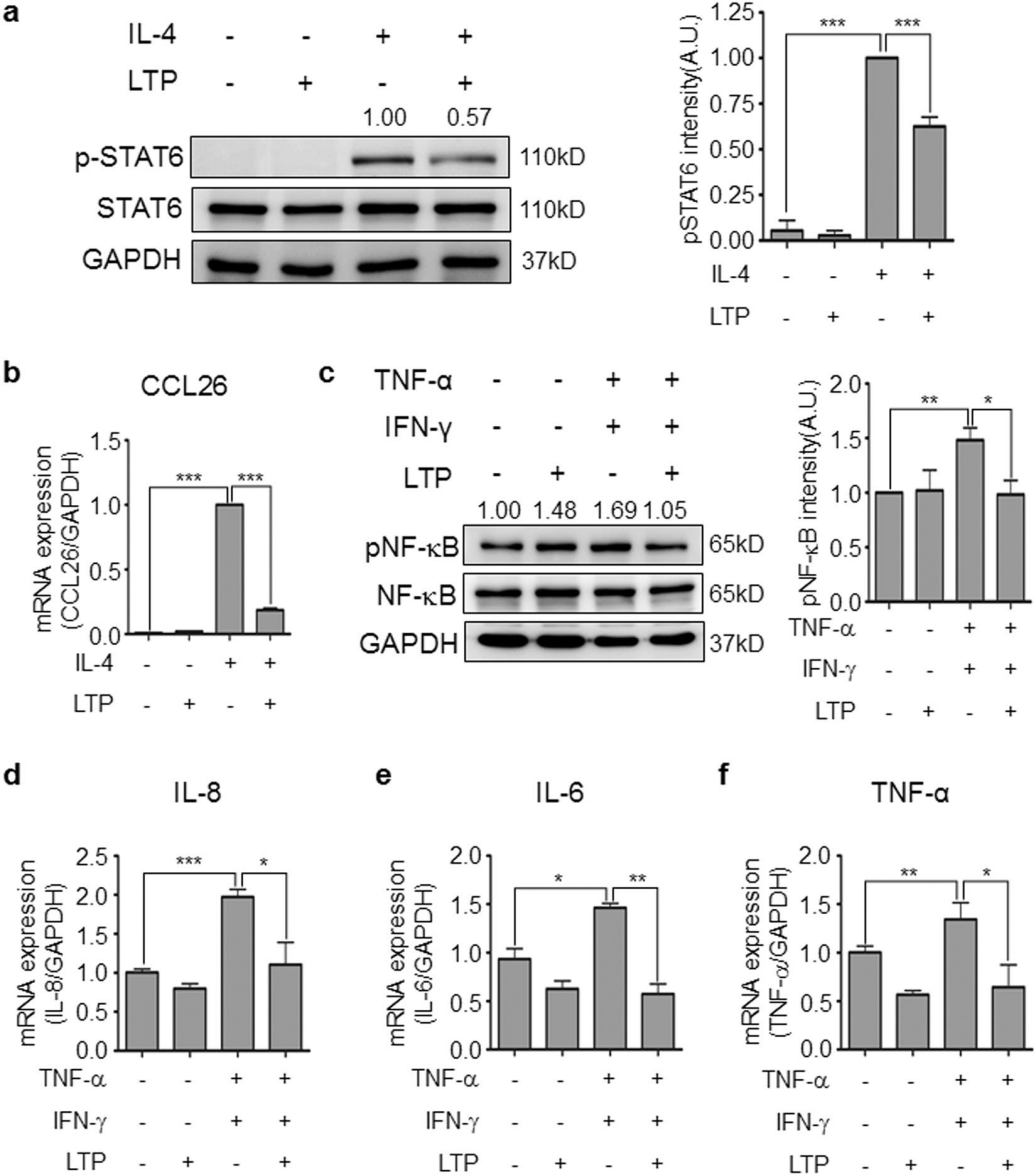
LTP treatment inhibits allergic inflammatory responses in HaCaT. (**a**) All Western blotting experiments were performed under the same condition. LTP treatment inhibited IL-4-induced STAT6 activation in HaCaT. IL-4 was stimulated with or without LTP for 15 min and the cells were collected for p-STAT6 detection using western blot analysis. The level of p-STAT6 was measured using ImageJ software. (**b**) LTP treatment inhibited IL-4-induced CCL26 expression in HaCaT. (**c**) All Western blotting experiments were performed under the same condition. LTP treatment inhibited TNF-α/IFN-γ-induced NF-κB activation in HaCaT. HaCaT cells were stimulated with TNF-α and IFN-γ for 15 min and the cells were harvested for p-NF-κB level using western blot analysis. The level of p-NF-κB was measured using ImageJ software. The expression of pro-inflammatory chemokine, (**d**) IL-8, and cytokine, (**e**) IL-6 and (**f**) TNF-α was detected in TNF-α/IFN-γ-stimulated HaCaT with or without LTP treatment using real-time PCR analysis. *P < 0.05, **P < 0.01, ***P < 0.001.

To confirm the anti-allergic inflammatory effect *in vitro*, we stimulated HaCaT, a keratinocyte cell line, with IL-4 and/or LTP and measured the level of STAT6 activation. The IL-4-stimulated group showed an increase in STAT6 activation. By contrast, the IL-4/LTP-treated group showed a decrease in STAT6 activation compared with IL-4 only stimulated group (Fig. 6a). In addition, LTP treatment inhibited IL-4-induced CCL26 expression, which mediated eosinophil recruitment (Fig. 6b). Expression of pro-inflammatory cytokines, such as TNF-α and IFN-γ, was also increased and stimulated other cells in chronic allergic responses. Thus, we determined whether LTP treatment inhibited TNF-α/IFN-γ-induced NF-κB activation and pro-inflammatory cytokine expression in HaCaT. Stimulation with TNF-α and IFN-γ induced NF-κB activation and increased the expression of pro-inflammatory cytokines, such as IL-8, IL-6, and TNF-α. However, LTP treatment suppressed the level of NF-κB activation (Fig. 6c) and pro-inflammatory cytokine expression (Fig. 6d–f) suggesting that plasma treatment might inhibit not only acute, but also chronic allergic responses.

## Discussion

Plasma medicine is an emerging field combining plasma physics and biomedical science for therapeutic application. Since the first clinical trials using NTP in 2010 to reduce bacteria in chronic wounds^21^, many experiments were performed and showed various effects including anti-cancer^22^, muscle regeneration^18^, wound healing^23^, and anti-inflammatory effect^20^. However, anti-allergic effect of plasma is not well-studied. In this study, we investigated anti-allergic effect of plasma in mice using mouse model of AD.

AD is a common allergic inflammatory skin disease and one of the major public health issues worldwide, with increasing incidence^24^. The etiology of AD involves immune system dysfunction, environmental exposures, and genetics. However, appropriate treatment has yet to be developed and the current treatments for AD only inhibit the disease symptoms without curing the disease^25^. Furthermore, current therapies have various side effects^26,27^. Thus, development of more effective therapies without side effects is necessary for the treatment of AD patients.

HDM is one of the most well-known allergens inducing AD^28^. Thus, we used HDM to induce AD-like skin inflammation in NC/Nga mice. Repeated application of HDM to the shaved skin of the mice induced symptoms of AD-like allergic skin inflammation, such as increased epidermal thickness, upregulation of cytokine and chemokine expression, and immune cell infiltration into the dermis^29–31^. Indeed, repeated application of HDM in NC/Nga mice induced AD-like skin inflammation, and N_2_-based NTP treatment ameliorated the AD-like allergic skin inflammation. NTP treatment decreased epidermal thickness (Fig. 2) and inhibited skin cell proliferation (Supplementary Fig. 6). Immune cell infiltration into skin tissue was also suppressed by LTP treatment (Fig. 2).

*In vitro* experiments are also showed that plasma treatment could inhibits skin inflammatory response in keratinocytes. Plasma treatment inhibits TNF-α/IFN-γ-induced NF-κB activation and translocation into the nucleus (Fig. 6c and Supplementary Fig. 5). IL-4 is an important cytokine for AD pathogenesis. Thus, we determined whether plasma treatment can suppress IL-4 signaling. Plasma treatment did inhibit IL-4-mediated STAT6 activation (Fig. 6a). In addition, plasma treatment in keratinocytes also suppressed STAT1 and STAT3 activation, which are involved in chronic allergic inflammation and autoimmune diseases, respectively, (Supplementary Fig. 9). He plasma can induce STAT1 and STAT3 activation^32^. However, in this study, we demonstrated that N_2_ plasma treatment inhibits cytokine-induced STAT1 and STAT3 activation (Supplementary Fig. 9). These results imply that the carrier gas might be important for plasma properties and that N_2_ plasma can inhibit STAT signaling, which is involved in inflammatory diseases.

We also investigated the effect of N_2_ plasma on ROS generation in keratinocytes because air plasma treatment increases ROS levels in cancer cells and eventually promotes cancer cell death^33^. In our study, N_2_ plasma treatment does not induce ROS generation in keratinocytes (Supplementary Fig. 7). The results imply that non-cancer cells may be able to regulate ROS levels after plasma treatment, and the results coincide with a previous report^34^. Furthermore, plasma treatment did not induce apoptosis in keratinocytes, although plasma can induce cell death in cancer cells^33^. Thus, we investigated whether N_2_ plasma can also induce apoptosis. TUNEL assay and western blot experiments showed that N_2_ plasma treatment did not induce apoptosis in keratinocytes (Supplementary Fig. 8). Taken together, our results suggest that N_2_ plasma inhibits inflammatory responses without cell death in keratinocytes.

A recent study demonstrated that Ar-based plasma inhibited DNCB-induced AD-like skin inflammation. However, the results showed that Ar-based plasma treatment was not effective in decreasing epidermal thickness^35^. By contrast, our results showed that treatment with N2-based NTP for AD-like skin inflammation reduced the epidermal thickness along with the inhibition of other allergic responses in HDM-induced AD-like skin inflammation in mice. To figure out the best condition for the anti-allergic effect of non-thermal plasma, more experiments with various plasmas generated from many different conditions should be performed.

In this study, we demonstrated that NTP can ameliorate AD-like skin inflammation in a mouse model of AD induced with HDM in NC/Nga mice. These results might suggest novel applications of NTP for the treatment of AD and other allergic diseases.

## Materials and Methods

### N_2_ plasma device

The N_2_ plasma device and the plasma generated from the device were described previously^18^. Briefly, N_2_ gas was used as a carrier gas of non-thermal atmospheric plasma. The gas flow rate, the input voltage, and the frequency were maintained at 10 L/min, 15 kV_p-p_, and 15 kHz, respectively. The optical emission spectra and current-voltage profile are shown in Supplementary Fig. 3. Plasma parameter, such as gas temperature, electron temperature and electron density are shown in Supplementary Fig. 4. We determined the presence of NO_2_ in the plasma-treated solution using a nitric oxide assay kit (Invitrogen) according to the manufacturer’s suggested procedures. Exposure of media to plasma for 1 min generated 60 µM of NO_2_.

### LTP generation and treatment

LTP was generated by the treatment of non-thermal N_2_ plasma into culture media (RPMI 1640 or DMEM) for 60 sec per ml at a 2 cm distant from the media (Fig. 6a). Cells were treated with LTP for 6 h for real-time PCR analysis. HaCaT cells were incubated with LTP for 15 min for pSTAT6, STAT6, pNF-κB and NF-κB detection.

### Cell line and reagents

The immortalized human keratinocyte cell line, HaCaT was grown as monolayer cultures in DMEM (Welgene, Daegu, Korea) supplemented with 10% fetal bovine serum (Gibco, Carlsbad CA), 1% penicillin and streptomycin (Gibco). Antibodies for STAT6, p-STAT6, NF-κB, p-NF-κB and GAPDH were purchased from Cell signaling technology (Danvers, MA). Anti-eosinophil peroxidase antibodies were purchased from Santa Cruz biotechnology. All recombinant cytokines were purchased from Peprotech (Rocky Hill, NJ).

### RNA isolation, complementary DNA synthesis, and quantitative real-time PCR analysis

Total RNA from cells or mouse skin tissue was isolated using TRIzol reagent (Gibco, Carlsbad, CA). Subsequently, the first strand of cDNA was synthesized with 1 µg total RNA using a ReverTra Ace qPCR RT Master Mix (TOYOBO, Japan). The primer sets from Qiagen (Hilden, Germany) were used for real-time PCR analysis and GAPDH transcript was used as an endogenous control. The amplification program consisted of 1 cycle at 95 °C for 10 min, followed by 45 cycles at 95 °C for 20 sec, 55 °C for 20 sec, and 72 for 20 sec.

### Western blot analysis

Western blot analysis was performed as previously described with minor modification^36^. Cells were lysed with RIPA buffer (Sigma Aldrich, St. Louis, MO, USA) containing a protease inhibitor cocktail and PhoSTOP (Roche Molecular Biochemicals, Basel, Switzerland). Ten micrograms of the protein lysate were loaded into 10% SDS-PAGE and transferred to PVDF membranes (Amersham, Arlington Heights, IL, USA). After transfer is finished, the membranes were incubated with blocking solution and then appropriate antibodies. The bands were visualized with Advanced ECL Western Blotting Detection Reagents (Amersham). GAPDH levels are used as a loading control.

### Mice

Six-week-old male NC/Nga mice were obtained from Orient Bio (Korea). These mice were housed in a controlled room with a 12:12-hour light-dark cycle and free access to laboratory chow and water. Seven to nine-week-old NC/Nga mice were used for animal experiments. The animal experiments in this study were approved by the Committee for Ethics in Animal Experiment of Ajou University School of Medicine and performed in accordance with the institution guidelines.

### HDM-induced AD in mice

AD was induced in NC/Nga mice by application of HDM as described previously with minor modification^37^. Briefly, dorsal hairs of the mice were removed completely. Next day, 200 μl of 4% SDS solution were used to dorsal skin to break the skin barrier followed by application of 100 mg of HDM containing cream (Biostir) every 4 days for 3 weeks. All the mice were sacrificed at a day after the final treatment. The mice were divided into four groups: non-treated control, NTP-treated only, Biostir-treated only and the topical application of NTP to Biostir-treated mice.

### Immunohistochemistry

The dorsal skins were fixed in 4% paraformaldehyde for 24 h and embedded in paraffin. The sections (4.5 μm) were obtained, stained with H&E or toluidine blue to monitor histological changes in the skin and mast cell recruitment, respectively. Eosinophil peroxidase (EPX) staining was carried out using a goat polyclonal anti-EPX antibody (Santa Cruz Biotechnology) to determine the recruitment of eosinophils.

### Mouse bone marrow-derived mast cells

BMMC were differentiated as previously described with minor changes^38^. Briefly, mouse bone marrow cells were collected from femurs of 7–8-week-old C57BL/6 mice and lineage-negative cells were purified using the lineage-negative cell isolation kit (Miltenyl Biotech, Germany) according to the manufacturer’s suggested protocols. The lineage-negative cells were cultured in RPMI 1640 medium supplemented with 10% FBS, 100 U/ml penicillin, and 100 µg/ml streptomycin in the presence of 10 ng/ml mIL-3 (PeproTech, USA) and 50 ng/ml mSCF (PeproTech, USA) for eight weeks at 37 °C in a humidified atmosphere with 5% CO_2_. The mast cell differentiation was determined by FACS analysis, and >94% of the cells were positive for CD117 and FcεRI, which are mast cell markers.

### Flow cytometry and intracellular cytokine analysis

Draining lymph nodes were isolated form mice and the lymph nodes were ground for single cell suspension. The cells were activated using anti-CD3 and anti-CD28 antibodies for 2 days and stained with anti-mouse IL-4, anti-mouse CD4 and anti-mouse CD3 antibodies (BioGems, Westlake Village, CA). Flow cytometry was performed on a FACSAria (BD Bioscience).

### Statistical analysis

Statistical comparisons between groups were performed using one-way analysis of variance (ANOVA) and Tukey’s post hoc tests. (*)*P* < 0.05, (**)*P* < 0.01 and (***)*P* < 0.001 were considered significant. All experiments were performed at least three times.

## Supplementary information

supplementary figures
