# Supplementary material for: Non-thermal plasma inhibits mast cell activation and ameliorates allergic skin inflammatory diseases in NC/Nga mice
Source: Sci Rep. 2019 Sep 18;9:13510. doi: 10.1038/s41598-019-49938-9 (PMC6751194; doi:10.1038/s41598-019-49938-9)
Supplement: Supplementary file 1 — supplementary figures [file 41598_2019_49938_MOESM1_ESM.pdf]

Non-thermal plasma inhibits mast cell activation and ameliorates allergic skin inflammatory diseases in NC/Nga mice.

Myung-Hoon Lee[1,2*], Yun Sang Lee[1*], Haeng Jun Kim[1,2], Chang Hak Han[1,2], Sung Un Kang[1] and Chul-Ho Kim[1,2,3]

[1]Department of Otolaryngology, School of Medicine, Ajou University, Suwon, Republic of Korea,

[2]Department of Molecular Science and Technology, Ajou University, Suwon, Republic of Korea,

[*] These authors are contributed this work equally. [3]Corresponding author: Chul-Ho Kim, M.D., Ph.D.

Supplementary figure 1

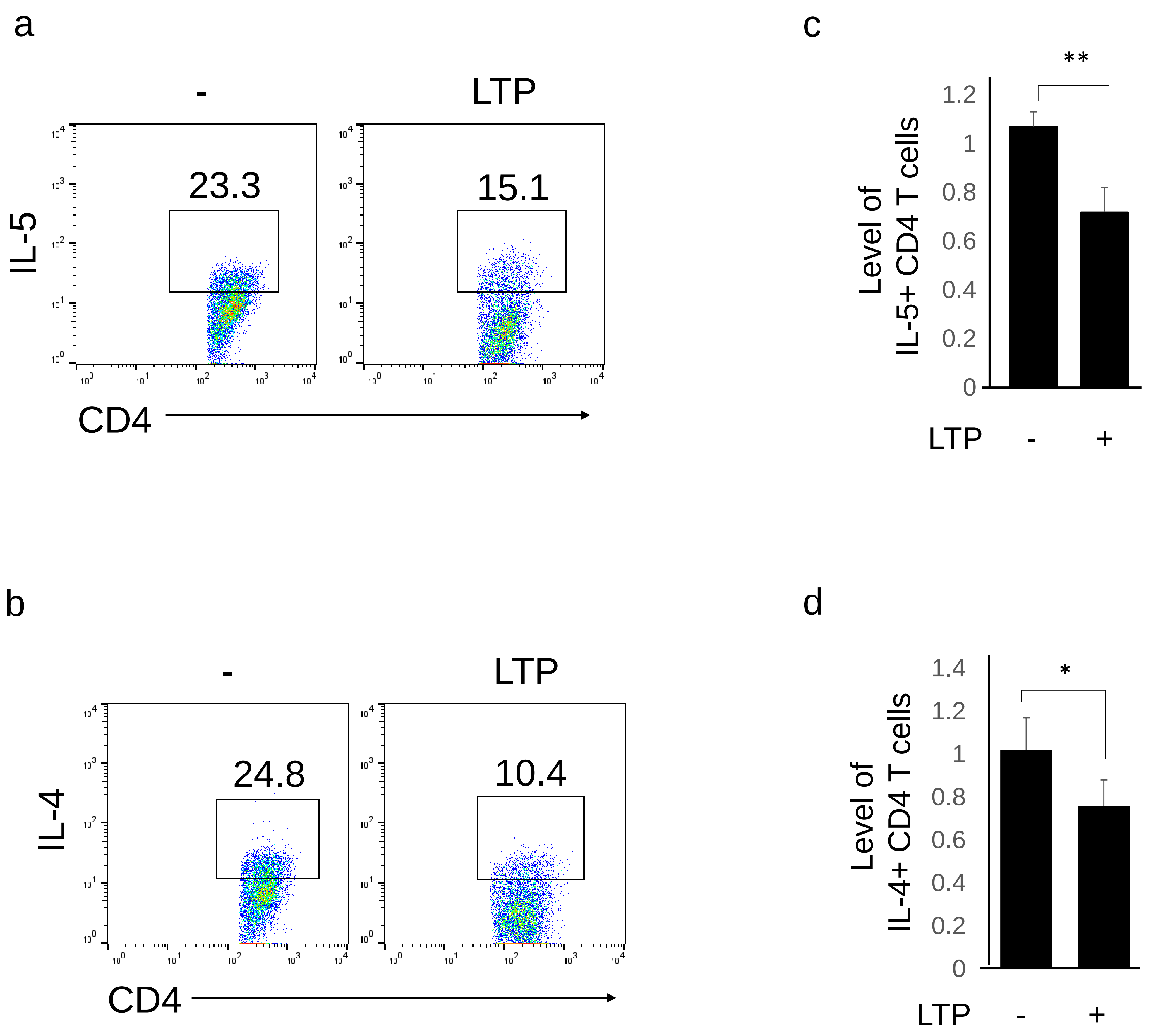

Supplementary figure 1. LTP treatment inhibits Th2 cell differentiation in vitro. Th2 cell was differentiated with anti-CD3 (10ug/ml), anti-CD28 (10ug/ml) antibodies and IL-4 (20ng/ml) in vitro with or without LTP.(a) Flow cytometry for IL-5 expressing CD4+ T cells (b) (d) Flow cytometry for IL-4 expressing CD4+ T cells. A representative example of three independent experiments is shown

Supplementary figure 2

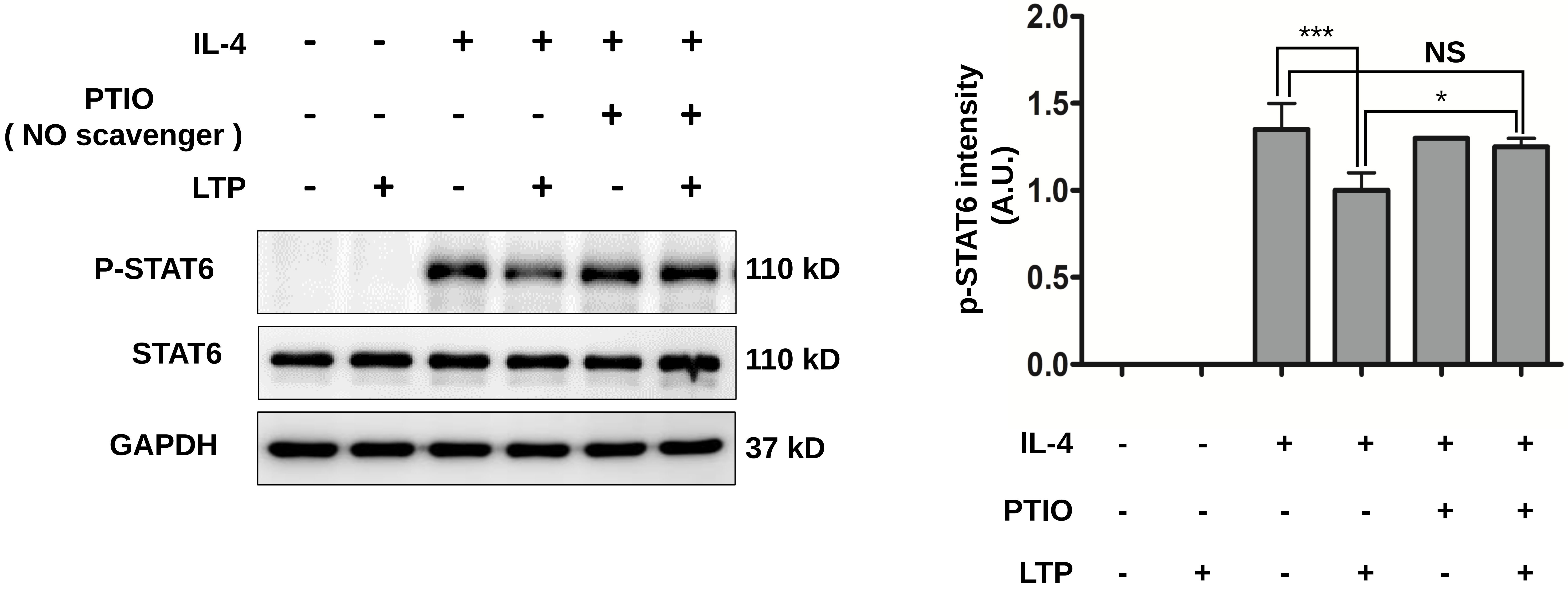

Supplementary figure 2. PTIO, a NO scavenger, inhibited IL-mediated STAT6 activation in epithelial cell, HaCaT. HaCaT cell was stimulated with an IL-4 (20ng/ml) for 15 min with or without LTP. Left: Treatment with IL-4 activated STAT6 and IL-4 stimulation with LTP inhibited the IL-4-induced STAT6 activation. However, PTIO treatment diminished the STAT6 activation inhibitory effect of LTP, implying that NO in LTP might be one of the components playing for anti-allergic inflammation. The figure is a representative example of three independent experiments. Right: The graph shows the band intensities of three independent western blot experiments.

Supplementary figure 3

a

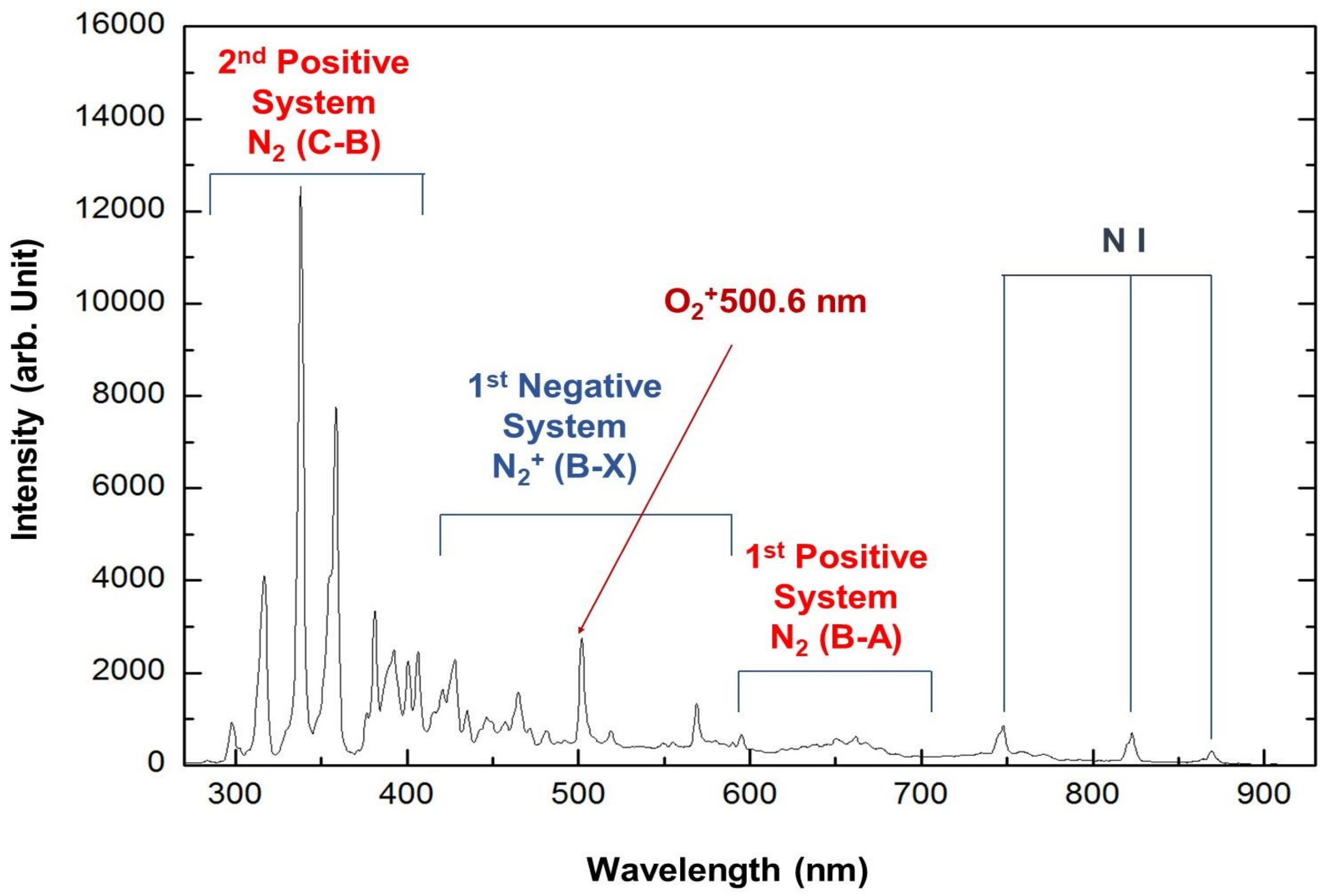

b

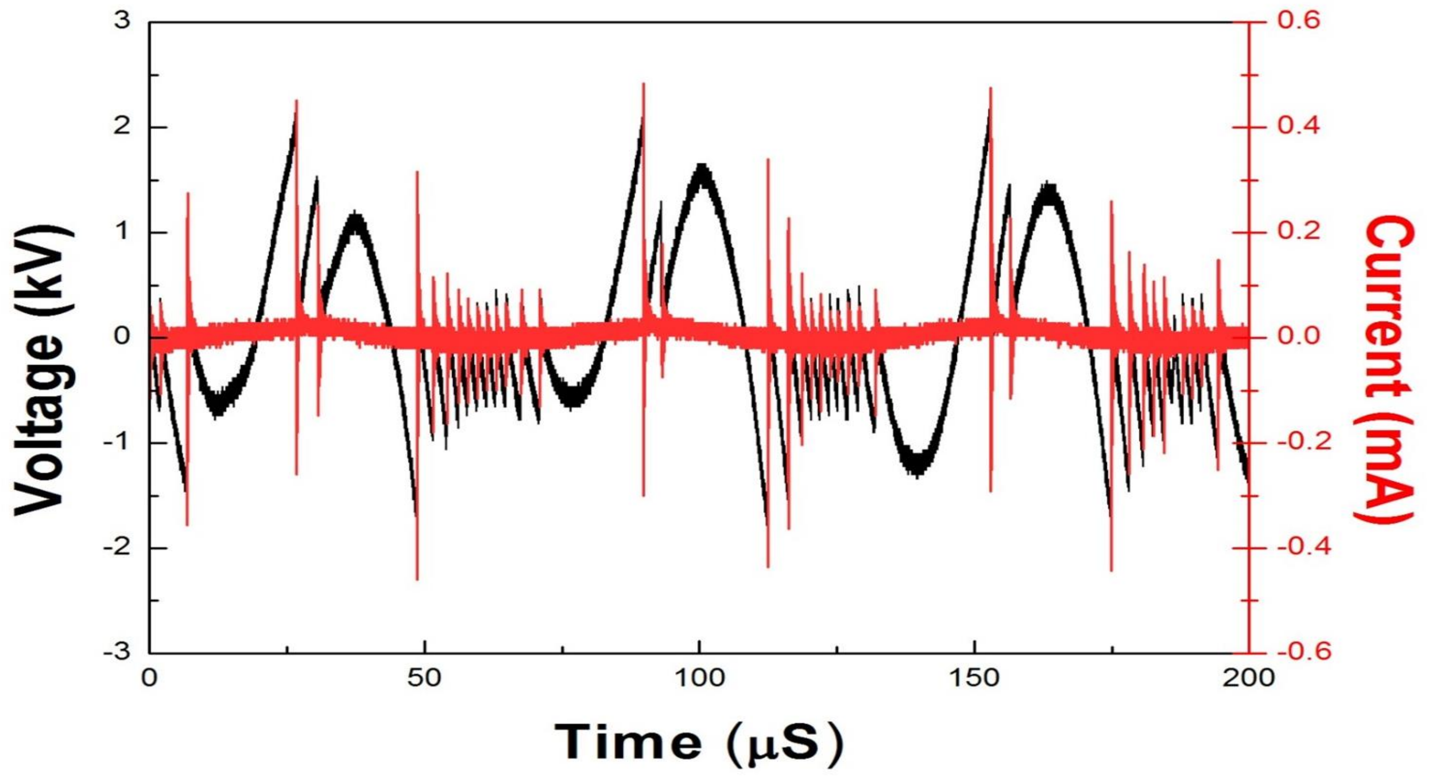

Supplementary figure 3. (a) Optical emission spectra profile of the plasma device. the emission spectra in range of 200 to 900nm for the plasma jet, operating 0.43 kV_{rms} and 46.4 mA_{rms}. The emission measurement was taken within the first 1 minute of the initiation of discharge. (b) current-voltage profile of the plasma device. The plasma jet used in this experiment, single micro discharge forms in the discharge gap every half voltage wave. Therefore, a single current peak is generated per half voltage wave when the voltage drops and the current increases. The root mean square value of the voltage (V_{rms}) and current (A_{rms}) were 0.43 kV and 46.4 mA, respectively.

Supplementary figure 4

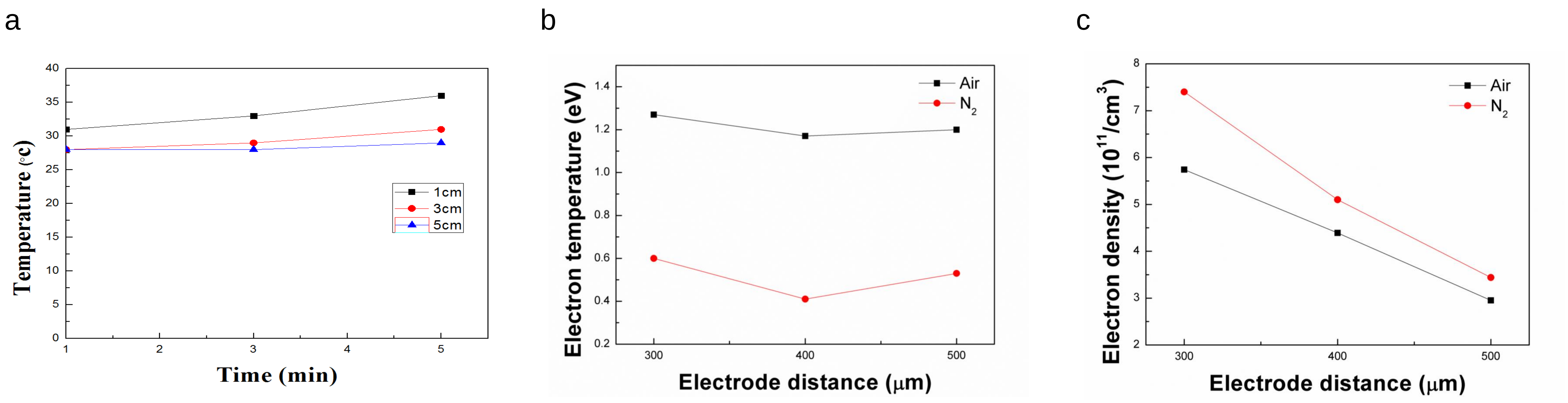

Supplementary figure 4. (a) Gas temperature (b) electron temperature (c) electron density of the N_2 plasma device.

Supplementary figure 5

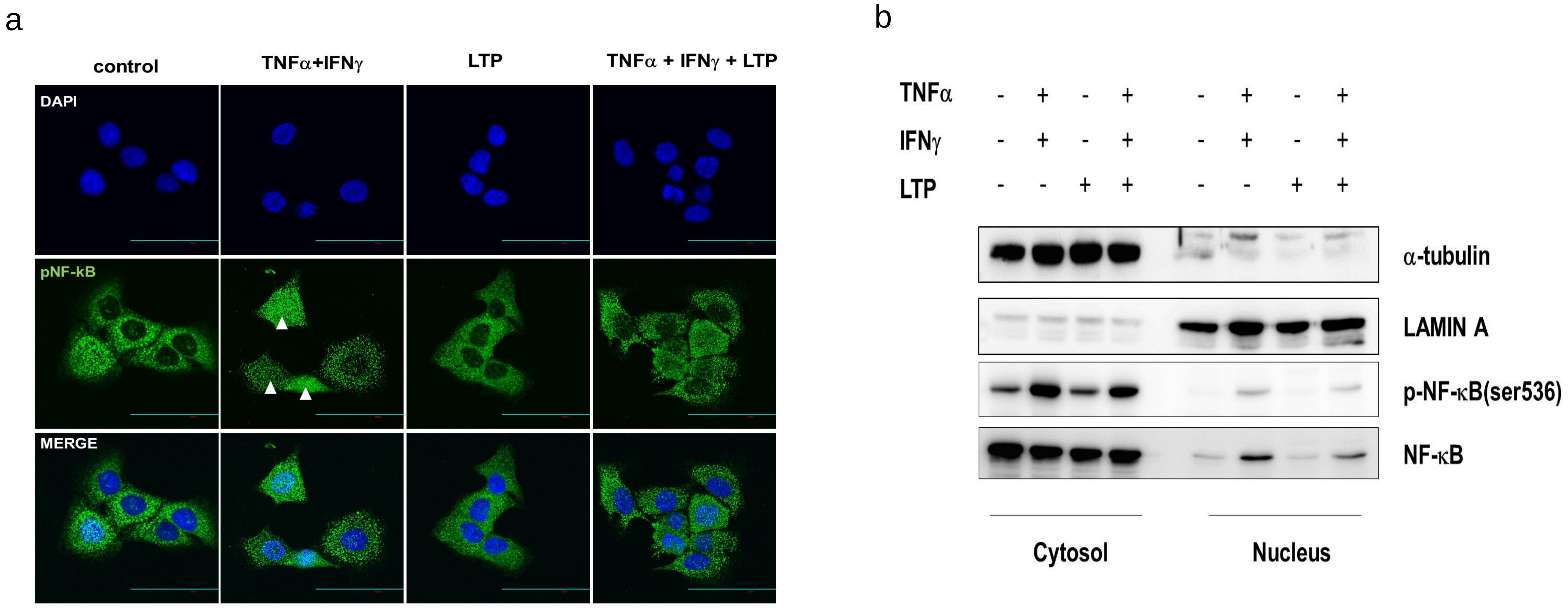

Supplementary figure5. LTP treatment inhibits cytokine-induced NF-κB translocation to the nucleus. (a) Immunofluorescence microscopy and (b) western blot analysis showed that LTP treatment inhibits activated NF-κB translocation to the nucleus

Supplementary figure 6

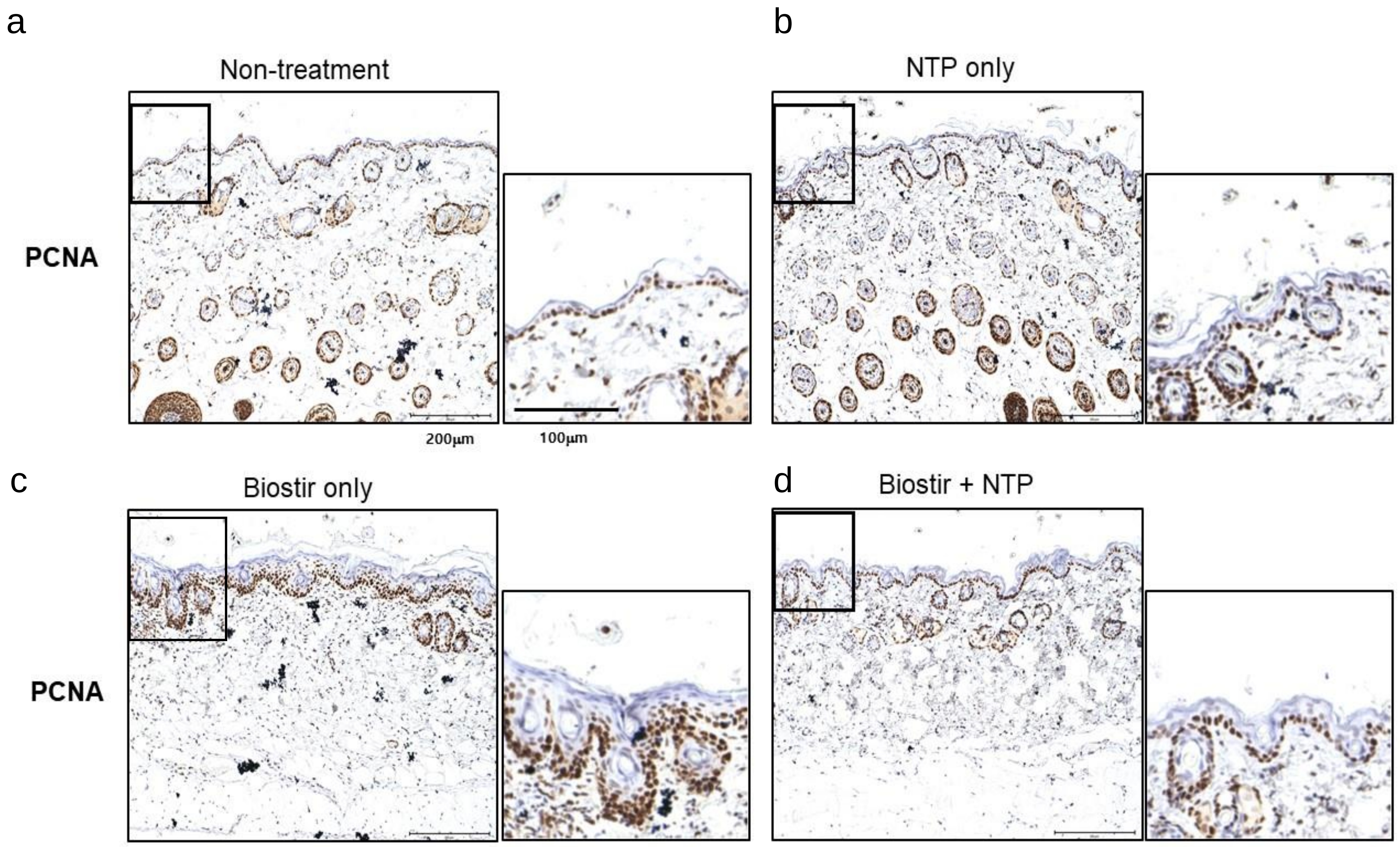

Supplementary figure 6. LTP treatment inhibits cell proliferation in house dust mite-treated mouse skin. (a) Non-treatment (b) NTP only treatment, (c) house dust mite only treatment (d) house dust mite and NTP treatment

Supplementary figure 7

a

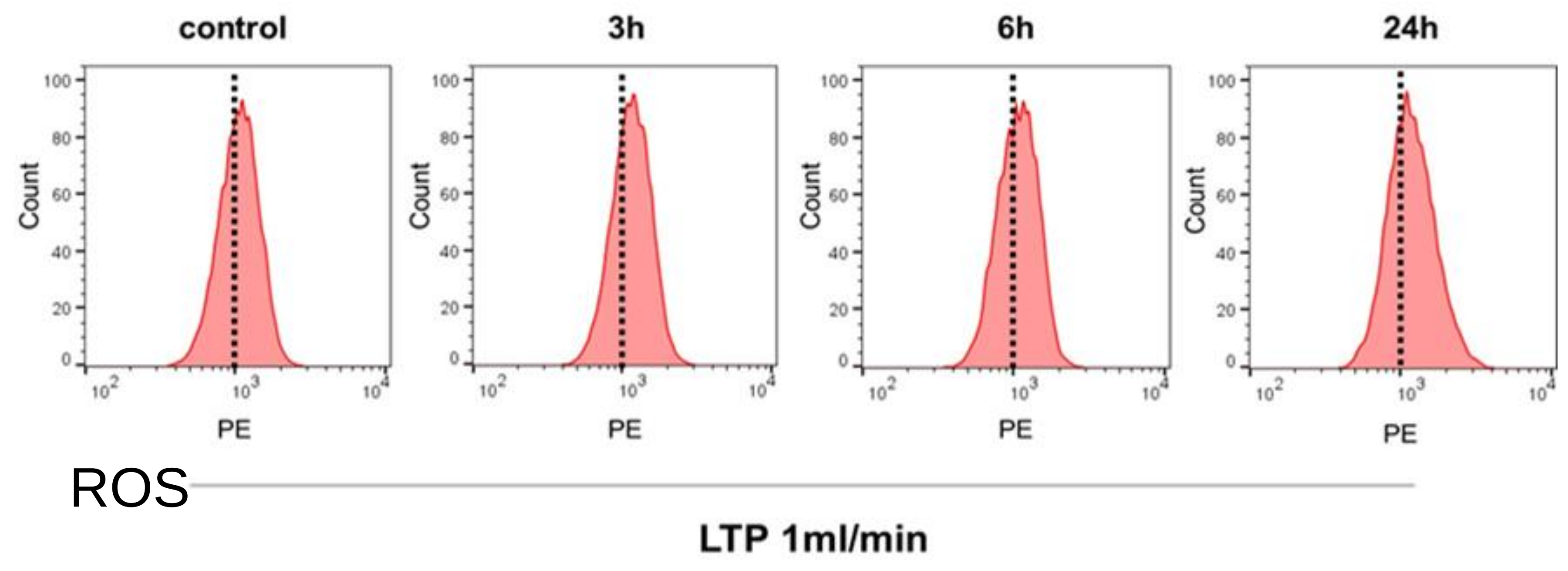

b

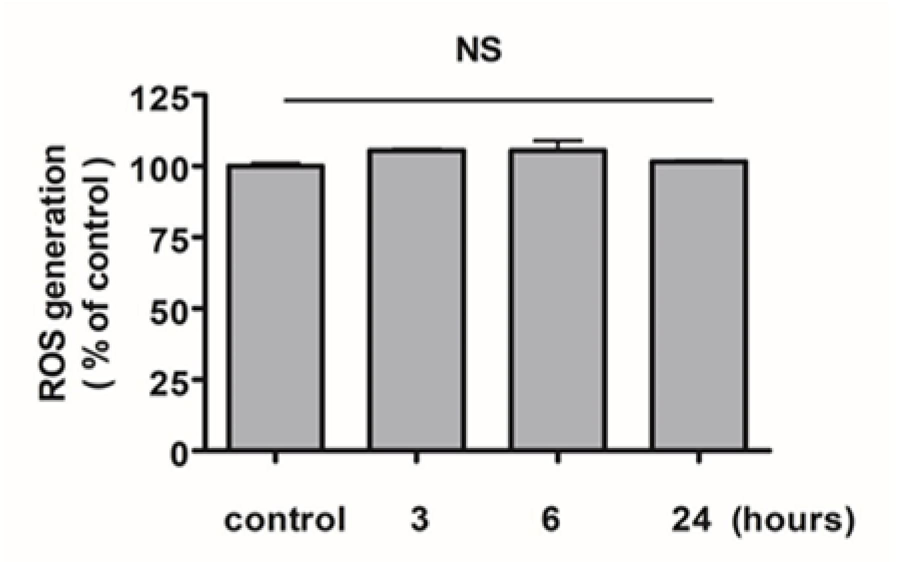

c

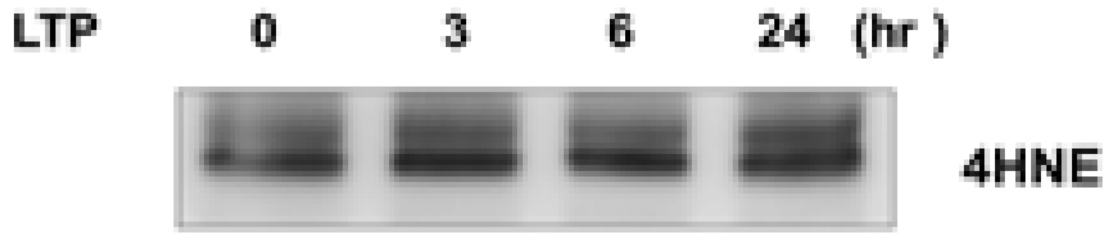

Supplementary figure 7. LTP treatment does not affect ROS generation in keratinocytes. (a) Flow cytometry for ROS detection (b) graph showing the relative amount of ROS , and (c) western blot analysis for 4HNE

Supplementary figure 8

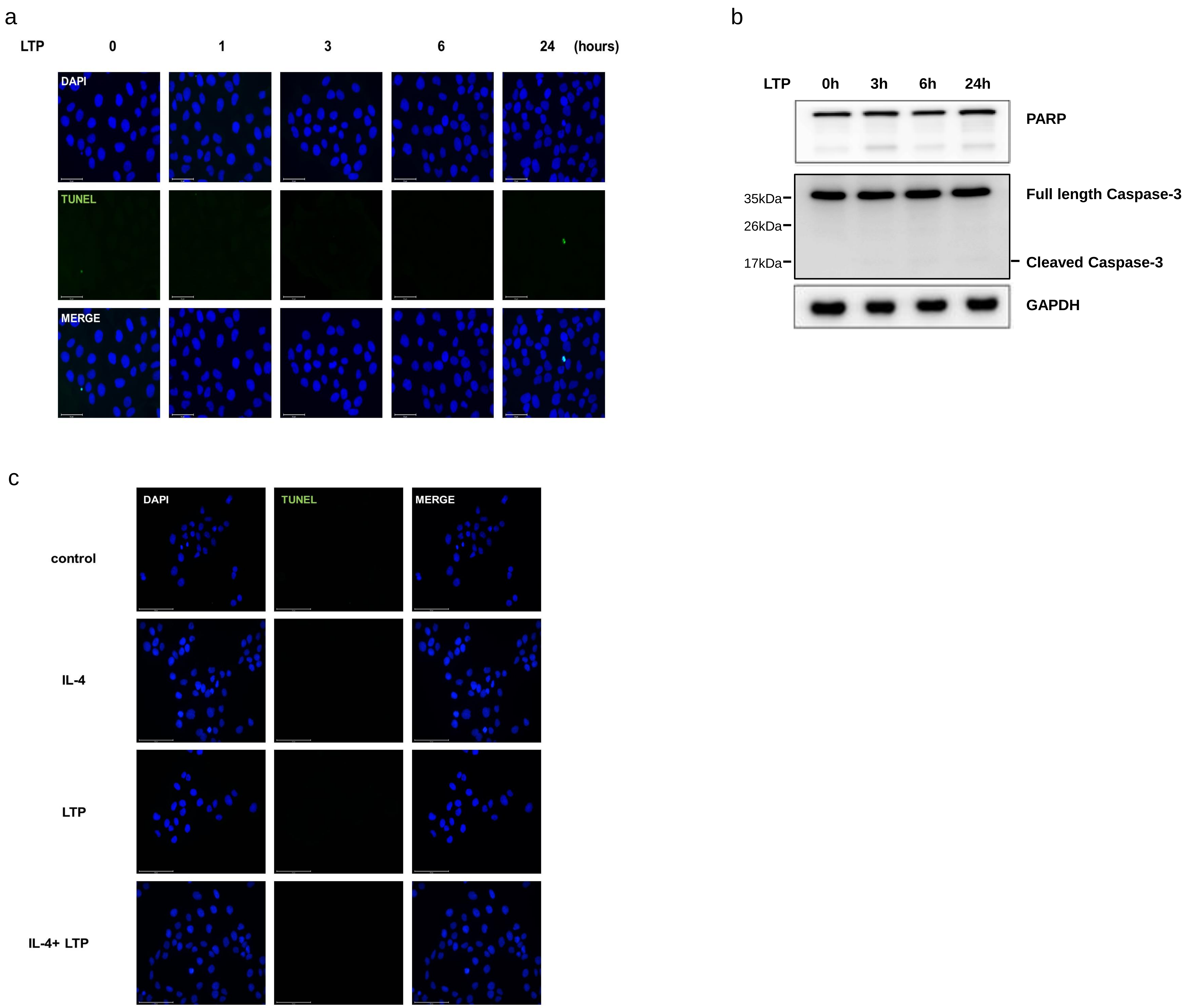

Supplementary figure 8. LTP treatment does not induce apoptosis in keratinocytes. (a and c) TUNEL assay and (b) western blot analysis shows that LTP treatment does not induce apoptosis in (a and b) keratinocyte and (c) IL-4-treated keratinocytes.

Supplementary figure 9

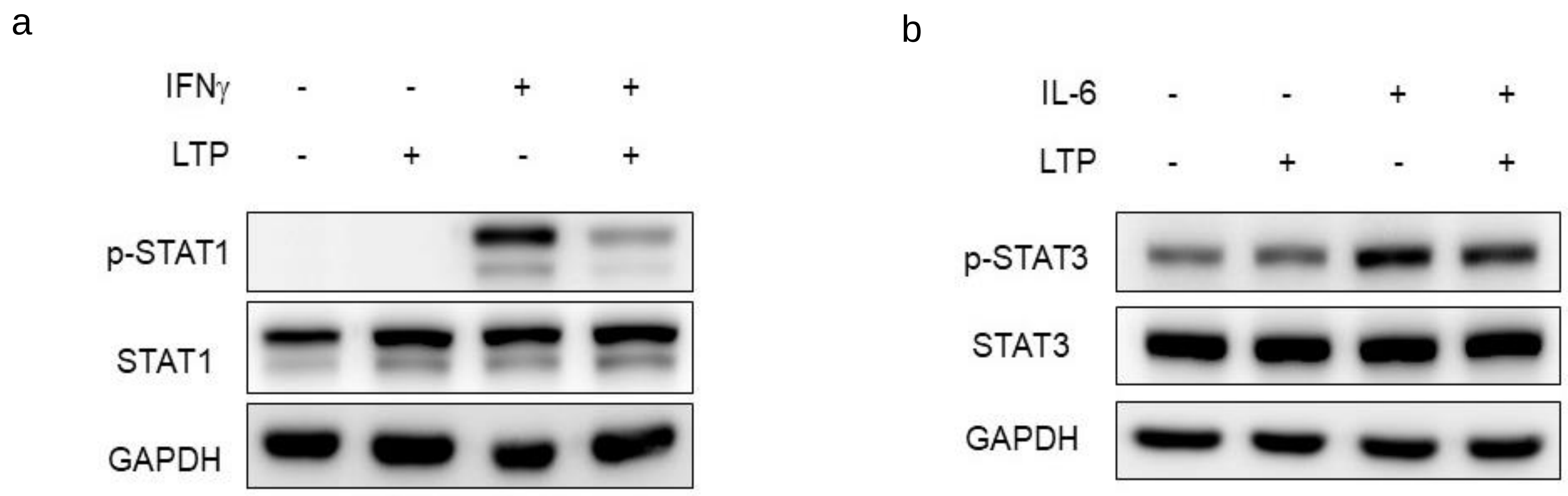

Supplementary figure 8. LTP treatment inhibits cytokine-induced STAT1 and STAT3 activation in keratinocytes. Western blot analysis shows that LTP treatment inhibits (a) IFNϒ- induced and (b) IL-6-induced STAT1 activation (pSTAT1) and STAT3 activation (pSTAT3), respectively in keratinocytes.
